# Trends in Population Blood Pressure and Prevalence, Awareness, Treatment, and Control of Hypertension among Middle-Aged and Older Adults in a Rural Area of Northwest China from 1982 to 2010

**DOI:** 10.1371/journal.pone.0061779

**Published:** 2013-04-16

**Authors:** Yaling Zhao, Hong Yan, Roger J. Marshall, Shaonong Dang, Ruihai Yang, Qiang Li, Xueying Qin

**Affiliations:** 1 Department of Public Health, Xi'an Jiaotong University College of Medicine, Xi’an, Shaanxi, P. R. China; 2 Section of Epidemiology and Biostatistics, School of Population Health, University of Auckland, Auckland, New Zealand; 3 Department of Cardiovascular Diseases, Hanzhong People’s Hospital, Hanzhong, Shaanxi, P. R. China; 4 Department of Epidemiology and Biostatistics, School of Public Health, Peking University, Beijing, P. R. China; Tulane School of Public Health and Tropical Medicine, United States of America

## Abstract

**Objectives:**

To assess trends in average blood pressure levels and prevalence, awareness, treatment, and control of hypertension among adults in a rural area of Northwest China, and to determine associated risk factors.

**Methods:**

Four cross-sectional population-based surveys were conducted between 1982 and 2010 among randomly selected adults in rural areas of Hanzhong, in Northwest China. Data on blood pressure, body mass index, family history of hypertension, and socio-demographic and lifestyle characteristics were collected in similar way by trained investigators in four surveys. Data of 8575 participants aged 35–64 years was analyzed. Averages and proportions were adjusted for age and sex.

**Results:**

Average blood pressure in the population has increased since 1982 from 76.9 mm Hg to 79.6 mm Hg in 2010 (diastolic) and from 120.9 to 129.7 mm Hg (systolic). Prevalence of hypertension increased from 18.4% in 1982 to 30.5% in 2010, and awareness of hypertension increased from 16.8% to 38.4% in 2010. Treatment of hypertension increased from 1.0% in 1982 to 17.4% in 2010, and control of hypertension increased from 0.1% in 1982 to 3.5% in 2010. All these gradients were statistically significant (P<0.01 for trend). Population blood pressure and prevalence, awareness and treatment of hypertension were positively associated with increasing age, body mass index and having family history of hypertension.

**Conclusions:**

Average blood pressure levels and the prevalence, awareness, treatment and control of hypertension among adults in rural areas of Hanzhong have increased since 1982. However, awareness, treatment and control rates remain low. Public health programs and practical strategies are required to improve prevention and control of hypertension in rural Northwest China. In particular, attention should be given to the elderly and obese, and to those with a family history of hypertension, while raising awareness and treatment among younger adults.

## Introduction

Hypertension (HTN) is one of the most important risk factors for coronary heart disease, stroke, and renal disease. Studies have demonstrated that lowering the elevated blood pressure (BP) may reduce the risk for morbidity and mortality due to cardiovascular diseases [1]. HTN is becoming an important global public health challenge [2]. It has been projected that by 2025, 29.2% of the world adult population will suffer from HTN and most of those affected will be found in developing countries [3]. In developed countries, population BP level and prevalence of HTN have lowered or remained stable over the past few decades, probably attributable to the improvement in detection, awareness and control of risk factors and better management of cases [4], [5], [6], [7], [8], [9], [10], [11]. However, the general trends of BP level and HTN in most developing countries, particularly in urban areas, are increasing and coming closer to developed countries, but rates of awareness, treatment, and control remain low [12], [13], [14], [15], [16]. This high prevalence and poor control are important factors in the rising epidemic of cardiovascular disease in developing countries [13].

China is experiencing rapid economic progress and rapid demographic and epidemiologic transitions. Non-communicable diseases, dominated by cardiovascular diseases such as obesity, diabetes mellitus and HTN have increased. Cardiovascular diseases have been the leading cause of both morbidity and mortality in China, responsible for one-third of all annual deaths [17]. The increased burden of cardiovascular diseases can be attributed, in part, to the rapid rise in hypertension. Two studies have described trends of HTN in China. One is a regional study examining trends between 1991 and 2007 in a rural area of Shandong Province, which is located in the east of China and is characterized by robust economic development [18]. The other is a partially representative national study, not including sites from Northwest China, from 1991 to 2009 [19]. However, little is known about the longitudinal trends of HTN in the rural areas of Northwest China, where is relatively poor and less developing compared with coastal, eastern and southern regions of China. To explore some of these issues, we studied the temporal changes in the prevalence, awareness, treatment, and control of HTN and in the population mean systolic blood pressure (SBP) and diastolic blood pressure (DBP) levels among a rural population in Northwest China over a 28 year period, from 1982 to 2010.

## Methods

### Study Setting and Participants

Four cross-sectional population-based epidemiological and risk factor surveys were conducted in 1982, 1998, 2004, and 2010, in the same rural areas of Hanzhong, Shaanxi Province, in Northwest China, using similar designs, to estimate the cardiovascular disease risk factors and epidemic of HTN among adults. For all the four surveys, the participants were restricted to people who have been living on the study sites for at least one year prior to the surveys. In 1982, individuals aged 30 to 64 years were invited to participate, while in 1998, individuals aged 30 to 84 years were invited, and in 2004, anyone aged 35 years and above was invited. In 2010, anyone aged 18 years and above was invited. To keep the same age range across surveys, only the participants aged 35 to 64 years, at the time of each survey, with complete data were included in the analysis.

### Data Collection

Data collection took place in villages of Hanzhong. Stratified randomized cluster sampling method was used. There are nine townships in the study area, and about 17 (15 to 36) villages in each township region. We stratified nine strata according to the township, that is, each township was a stratum and one or two villages (clusters) were randomly chosen, separately for each survey, from each township. Using residential registration data, all the available and eligible adults in the chosen villages were informed of and invited to participate in the survey several days before the surveys. 200 to 400 adults (the number varied through the four surveys according to the pre-determined sample size of each survey) who consented and came to the clinic of the village doctor, where the interview and physician examination were conducted, on the survey day were chosen as subjects of the surveys. Data was collected by trained doctors and nurses from Hanzhong People’s Hospital, but in 2010, several graduate students from Xi'an Jiaotong University College of Medicine participated in data collection. Information was collected by interview on age, sex, level of education, marital status, life habits such as smoking and drinking, family history of HTN, history of HTN, and use of antihypertensive medications. BP, height, and weight were measured during the physician examination. In the surveys, the participants were asked about monthly alcohol consumption, including grape wine, rice wine, beer and liquor within the recent year. The participant who consumed alcohol less than once per month was defined as non-drinker and participant who consumed alcohol equal to or more than once per month was defined as drinker, i.e. current alcohol drinking. Weight and height were measured with participants standing without shoes or heavy outer garments, from which body mass index (BMI) was calculated. Using World Health Organization criteria, BMI was categorized into four groups as underweight (BMI<18.5), normal weight (18.5≤BMI<25.0), overweight (25.0≤BMI<30.0) and obese (BMI≥30.0). Age at interview was categorized in five year intervals. Education status was classed into illiterate, elementary, middle school, and high school and above.

BP was measured, after the subject had rested for at least 5 min, using a standard mercury sphygmomanometer with the participant in the sitting position. BP values were recorded to the nearest 2 mm Hg. The mean of two or three readings was used as BP value; two in the 2010 survey and three in the earlier surveys. As categorized by JNC-7, we defined pre-hypertension (pre-HTN) as a mean SBP of 120 to 139 mm Hg or a mean DBP of 80 to 89 mmHg, and HTN as a mean SBP≥140 mm Hg, and/or a mean DBP≥90 mm Hg, and/or self-reported current treatment with antihypertensive medication [20]. Awareness of HTN was defined as self-report of previous diagnosis of HTN by a health care professional. Treatment of HTN was defined as self-reported current use of antihypertensive medication. Control of HTN was defined as hypertensive participants’ SBP<140 mm Hg and DBP<90 mm Hg. And control of HTN (SBP<140 mm Hg and DBP<90 mm Hg) among hypertensive participants who treated their HTN with drugs, that is, control in treatment, was also analyzed.

### Statistical Analyses

The Complex Samples Procedure of SPSS 13.0 for Windows (SPSS Inc., Chicago, Illinois, USA) was used for statistical analyses, accounting for township strata and village clusters. All statistical tests were two-tailed, and statistical significance was set at P<0.05. Continuous variables were presented as mean values. Categorical variables were presented as frequencies. Since age and sex distributions in the four surveys varied, overall means of BP and prevalence, awareness, treatment, control of HTN of each year were adjusted for age and/or sex, according to the 2000 Chinese National Census population distribution except for age-specific and/or sex-specific means and percentages. Differences between means were compared using General Linear Models. Chi-square tests were used to compare frequencies. Trends in means and the estimated percentages were assessed with General Linear Models (continuous outcomes) and Logistic Regression Models (dichotomous outcomes). General Linear Models (continuous outcomes) and Logistic Regression Models (dichotomous outcomes) were also used to evaluate the association between BP, prevalence, awareness, treatment, and control of HTN and associated risk factors. Because there was no data on marital status, education level and family history of HTN in the 1982’s survey, the analyses for associated risk factors were conducted using data from 1998 to 2010.

### Ethics Statement

The study complied with the Declaration of Helsinki and was reviewed and approved by the Ethics Committee of Xi’an Jiaotong University College of Medicine and written informed consent had been obtained from the study participants.

## Results

### General Characteristics of the Study Population

There were 3730, 1016, 1353, and 2476 subjects aged 35–64 years of age, respectively, in the 1982, 1998, 2004, and 2010 surveys. The socio-demographic characteristics, lifestyle factors, BMI, and family history of HTN of these participants are shown in Table 1.

**Table 1:** Characteristics of the population in the study.

Characteristics	1982	1998	2004	2010	P value
Number of participants, n (%)					
Overall	3730 (100.0)	1016 (100.0)	1353 (100.0)	2476 (100.0)	
Men	1646 (44.1)	482 (47.4)	536 (39.6)	830 (33.5)	<0.001*
Women	2084 (55.9)	534 (52.6)	817 (60.4)	1646 (66.5)	
Age, years, mean (SE)	45.8 (0.1)	47.9 (0.2)	51.2 (1.3)	50.7 (0.4)	<0.001*
Marital status (%)					0.003
Married	–	98.0	93.2	94.3	
Unmarried	–	0.6	0.4	0.4	
Divorced, separate, widowed	–	1.4	6.4	5.3	
Level of education (%)					<0.001
Illiterate	–	17.1	15.8	11.6	
Elementary	–	36.1	41.6	33.0	
Middle School	–	32.7	32.6	44.3	
High School and above	–	14.1	10.0	11.1	
Current cigarette smoking (%)	36.3	35.5	30.6	26.5	<0.001*
Current alcohol drinking (%)	33.8	30.1	32.9	33.0	0.507*
Family history of HTN (%)	–	34.5	35.4	34.1	0.551*
BMI, kg/m^2^, mean (SE)	21.0 (0.1)	22.5 (0.4)	23.0 (0.2)	23.2 (0.1)	<0.001*
Underweight (BMI<18.5, %)	7.5	6.7	3.7	5.3	<0.001*
Normal (18.5≤BMI<25.0, %)	90.1	75.5	73.1	71.0	<0.001*
Overweight (25≤BMI<30.0, %)	2.3	17.0	21.6	22.4	<0.001*
Obesity (BMI≥30.0, %)	0.1	0.8	1.6	1.3	<0.001*

HTN, hypertension; BMI, body mass index.

–Without data.

* P value for trend.

The mean age and the distribution of sex, marital status and level of education were different in the four surveys (all P<0.01). Percentage of current smokers decreased from 36.3% to 26.5% from 1982 to 2010 (P<0.001 for trend), whereas drinkers remained stable at around 33% (P = 0.507 for trend). People with family history of HTN remained stable at around 34% (P = 0.551 for trend from 1998 to 2010, no data about family history of HTN in 1982). Mean BMI was 21.0 in 1982 and 23.2 in 2010, and had an overall increase from 1982 to 2010 (P<0.001 for trend). Rates of underweight and normal weight decreased from 7.5% and 90.1%, respectively, in 1982 to 5.3% and 71.0%, respectively, in 2010 (both P<0.001 for trend). In contrast, rates of overweight and obesity increased from 2.3% and 0.1% to 22.4% and 1.3%, respectively, over the past 28 years (both P<0.001 for trend).

### Changes in BP

**Figure 1:**
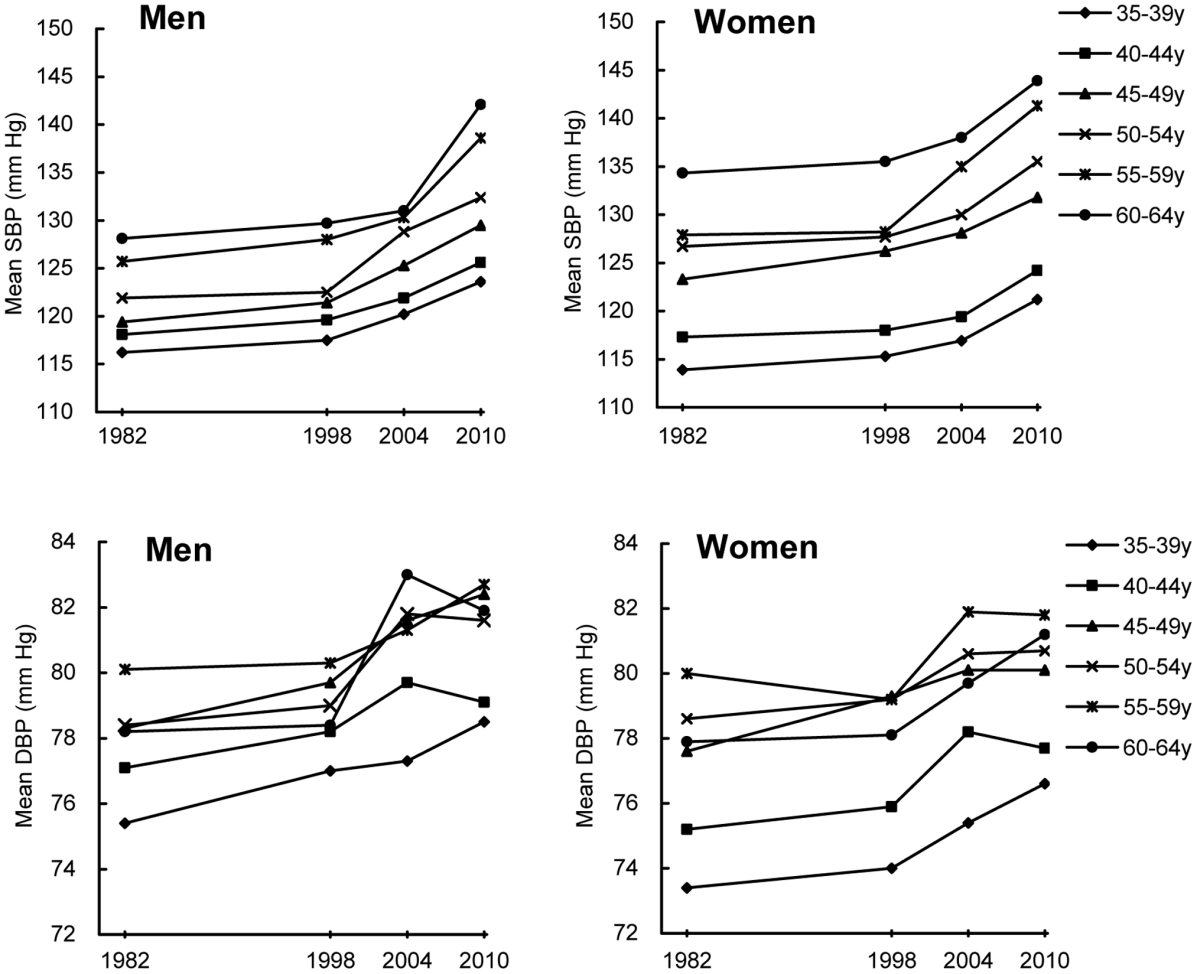
Trends in mean SBP and DBP among adults in different sex and age group, 1982 to 2010.

Mean SBP and DBP from 1982 to 2010 are shown in Table 2. Overall, both mean SBP and DBP values increased significantly from 1982 to 2010 (P<0.001 for trend). Age- and sex- adjusted mean SBP and DBP were 120.9 mm Hg and 76.9 mm Hg, respectively, in 1982, and 129.7 mm Hg and 79.6 mm Hg, respectively, in 2010. Similar significantly increasing trends in mean levels of SBP and DBP were observed in all subgroups defined by age and sex (all P<0.01 for trend; Figure 1). Mean SBP values increased with increasing age in both men and women (P<0.001 for trend), and mean SBP values were higher in men than women before age 45 and lower than women at age 45 to 64 (all P<0.01). Mean DBP values increased with age until 59 years (P<0.001 for trend), and mean DBP values among men and women aged 60–64 years old were decreased, but the decrease was not significant (P = 0.079 for comparison with 50–59 years olds); between age 35–64, men had higher mean DBP values than women, but the difference was not significant among men and women 50 and above years old (all P>0.05).

**Table 2:** Mean SBP and DBP (mm Hg) for adults aged 35 to 64 years from 1982 to 2010.

Characteristics	1982		1998		2004		2010		P valuefor trend
	n	mean,95% CI	n	mean,95% CI	n	mean,95% CI	n	mean,95% CI	
SBP									
Overall*	3730	120.9 (119.0–123.0)	1016	122.4 (118.0–126.9)	1353	125.0 (121.2–128.8)	2476	129.7 (126.0–133.4)	<0.001
Men†	1646	120.1 (118.3–122.2)	482	121.7 (118.3–125.2)	536	124.7 (120.6–128.9)	830	129.5 (125.8–133.2)	<0.001
Women†	2084	121.7 (119.8–123.8)	534	123.1 (117.6–128.7)	817	125.2 (121.7–128.7)	1646	129.9 (126.3–133.5)	<0.001
DBP									
Overall*	3730	76.9 (75.5–78.6)	1016	77.8 (74.6–81.1)	1353	79.1 (76.8–82.4)	2476	79.6 (77.3–81.8)	<0.001
Men†	1646	77.4 (75.9–79.1)	482	78.5 (75.8–81.3)	536	79.9 (77.1–83.7)	830	80.4 (78.0–82.7)	<0.001
Women†	2084	76.4 (74.9–78.1)	534	77.1 (73.4–80.9)	817	78.2 (76.5–81.0)	1646	78.7 (76.6–80.8)	<0.001

SBP, systolic blood pressure; DBP, diastolic blood pressure; 95% CI, 95% confidence interval.

* Age- and sex- adjusted mean SBP or DBP values.

† Age- adjusted mean SBP or DBP values for men or women. Adjustment was conducted with the 2000 Chinese National Census population by the direct method.

### Changes in Prevalence of HTN, Pre-HTN and Normotensive

**Figure 2:**
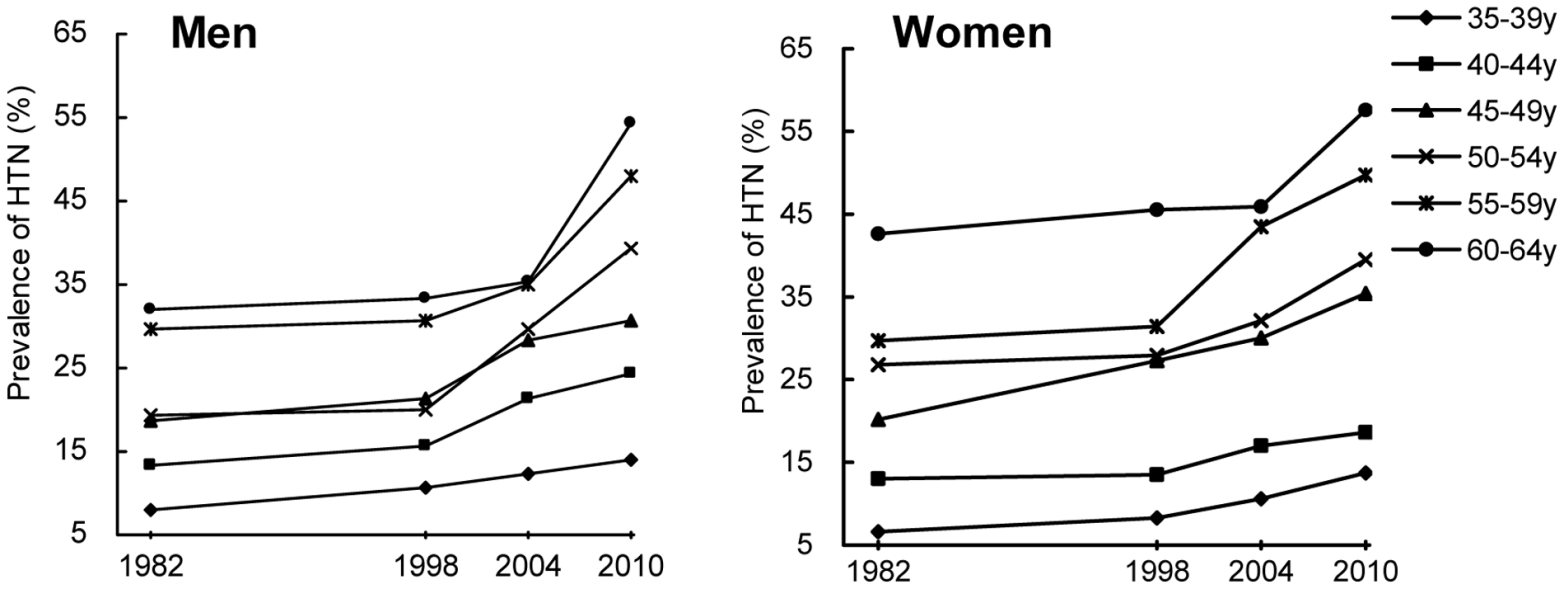
Trends in prevalence of HTN among adults in different sex and age group, 1982 to 2010.

**Figure 3:**
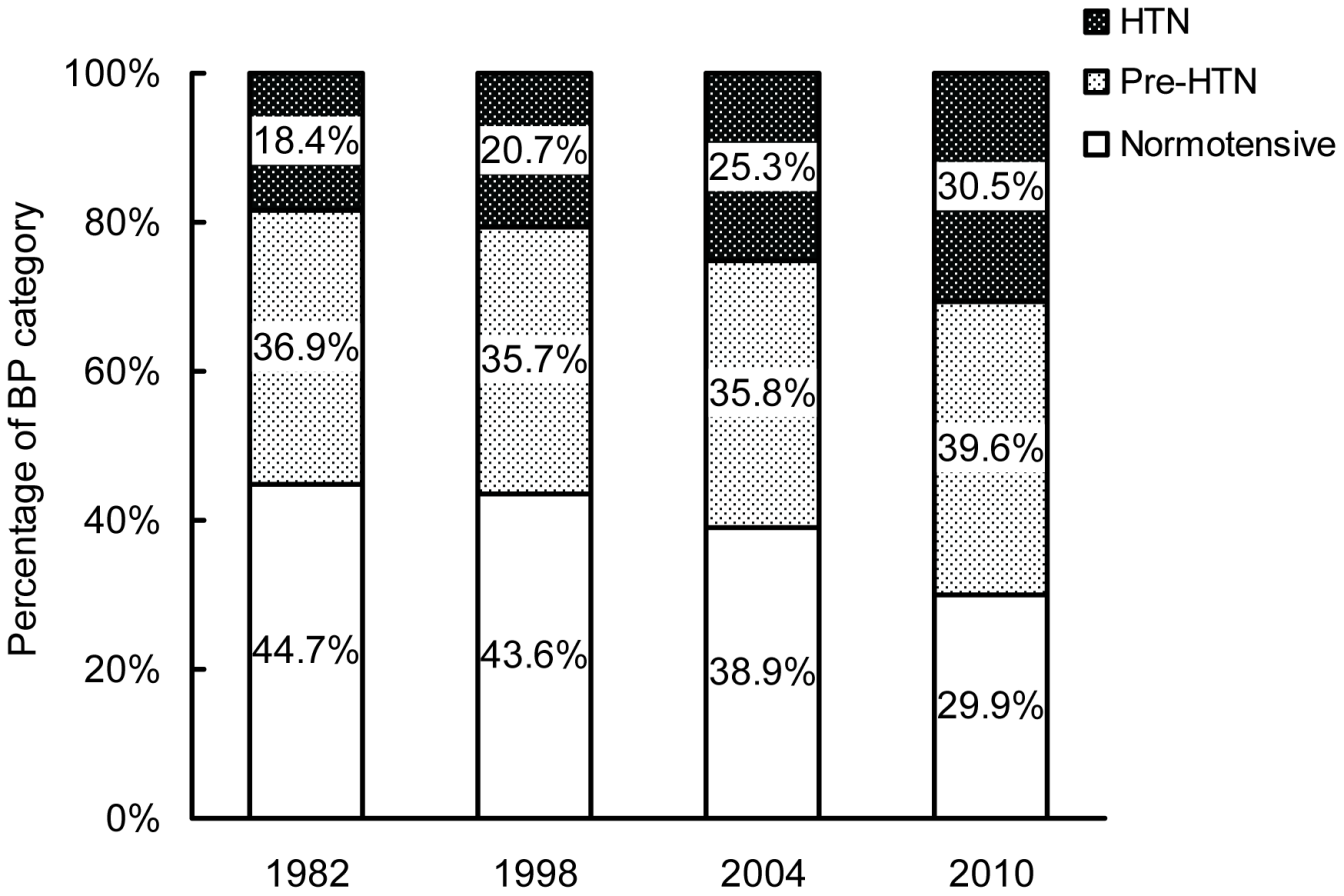
Change in distribution of BP categories, 1982 to 2010.

The prevalence of HTN, pre-HTN and normotensive among adults aged 35–64 years from 1982 to 2010 is shown in Table 3. The prevalence of HTN significantly increased over the 28-year period, both in men and women among all age subgroups (all P<0.001 for trend; Figure 2). Age- and sex- adjusted prevalence of HTN increased from 18.4% in 1982 to 30.5% in 2010. Age-adjusted prevalence of HTN in men increased from 17.4% in 1982 to 30.6% in 2010. And that in women increased from 19.4% in 1982 to 30.5% in 2010. And the prevalence rates were higher in men than women before age 45, and were lower in men than women between ages 45 to 64, however, the difference was not significant (all P>0.05). Prevalence of HTN increased with increasing age in both men and women (P<0.001 for trend). The age- and sex- adjusted pre-HTN prevalence were 36.9% in 1982 and 39.6% in 2010, with no significant change from 1982 to 2010 (P = 0.745 for trend). Prevalence of pre-HTN showed no different between men and women or between different age groups (all P>0.05). Percentages of people with normal BP levels showed a significant decrease from 1982 to 2010 in both sex and all age groups (all P<0.001 for trend). Overall, percentage of normotensive decreased from 44.7% in 1982 to 29.9% in 2010 (Figure 3).

**Table 3:** Prevalence of HTN, pre-HTN and normotensive for adults aged 35 to 64 years from 1982 to 2010.

Characteristics	1982		1998		2004		2010		P value for trend
	n/N	% (95% CI)	n/N	% (95% CI)	n/N	% (95% CI)	n/N	% (95% CI)	
HTN									
Overall*	651/3730	18.4 (13.6–23.6)	219/1016	20.7 (13.6–28.2)	423/1353	25.3 (16.9–36.1)	916/2476	30.5 (24.1–37.9)	<0.001
Men†	286/1646	17.4 (12.6–22.6)	96/482	19.4 (14.2–24.9)	156/536	26.2 (16.2–38.9)	314/830	30.6 (22.7–39.5)	<0.001
Women†	365/2084	19.4 (14.6–24.6)	123/534	22.0 (12.9–31.8)	267/817	24.3 (17.7–33.1)	602/1646	30.5 (25.7–36.1)	<0.001
Pre-HTN									
Overall*	1366/3730	36.9 (31.5–43.0)	382/1016	35.7 (23.9–47.4)	488/1353	35.8 (25.8–47.4)	905/2476	39.6 (33.3–46.9)	0.745
Men†	640/1646	38.8 (33.1–44.9)	189/482	38.6 (27.7–49.8)	206/536	38.2 (27.1–51.0)	317/830	42.2 (35.1–49.9)	0.491
Women†	726/2084	34.9 (29.8–41.0)	193/534	32.5 (19.7–44.9)	282/817	33.2 (24.5–43.4)	588/1646	36.9 (31.5–43.8)	0.603
Normotensive									
Overall*	1713/3730	44.7 (38.1–50.9)	415/1016	43.6 (30.9–57.1)	442/1353	38.9 (30.2–50.4)	655/2476	29.9 (23.0–38.0)	<0.001
Men†	720/1646	43.8 (37.0–50.1)	197/482	41.9 (29.7–53.7)	174/536	35.6 (24.7–48.3)	199/830	27.2 (19.8–36.1)	0.001
Women†	993/2084	45.7 (39.3–51.8)	218/534	45.5 (32.1–60.7)	268/817	42.5 (36.1–52.6)	456/1646	32.7 (26.4–40.0)	<0.001

HTN, hypertension; pre-HTN, pre-hypertension; 95% CI, 95% confidence interval.

* Age- and sex- adjusted percentages.

† Age- adjusted percentages for men or women. Adjustment was conducted with the 2000 Chinese National Census population by the direct method.

### Changes in Awareness, Treatment, and Control of HTN

**Figure 4:**
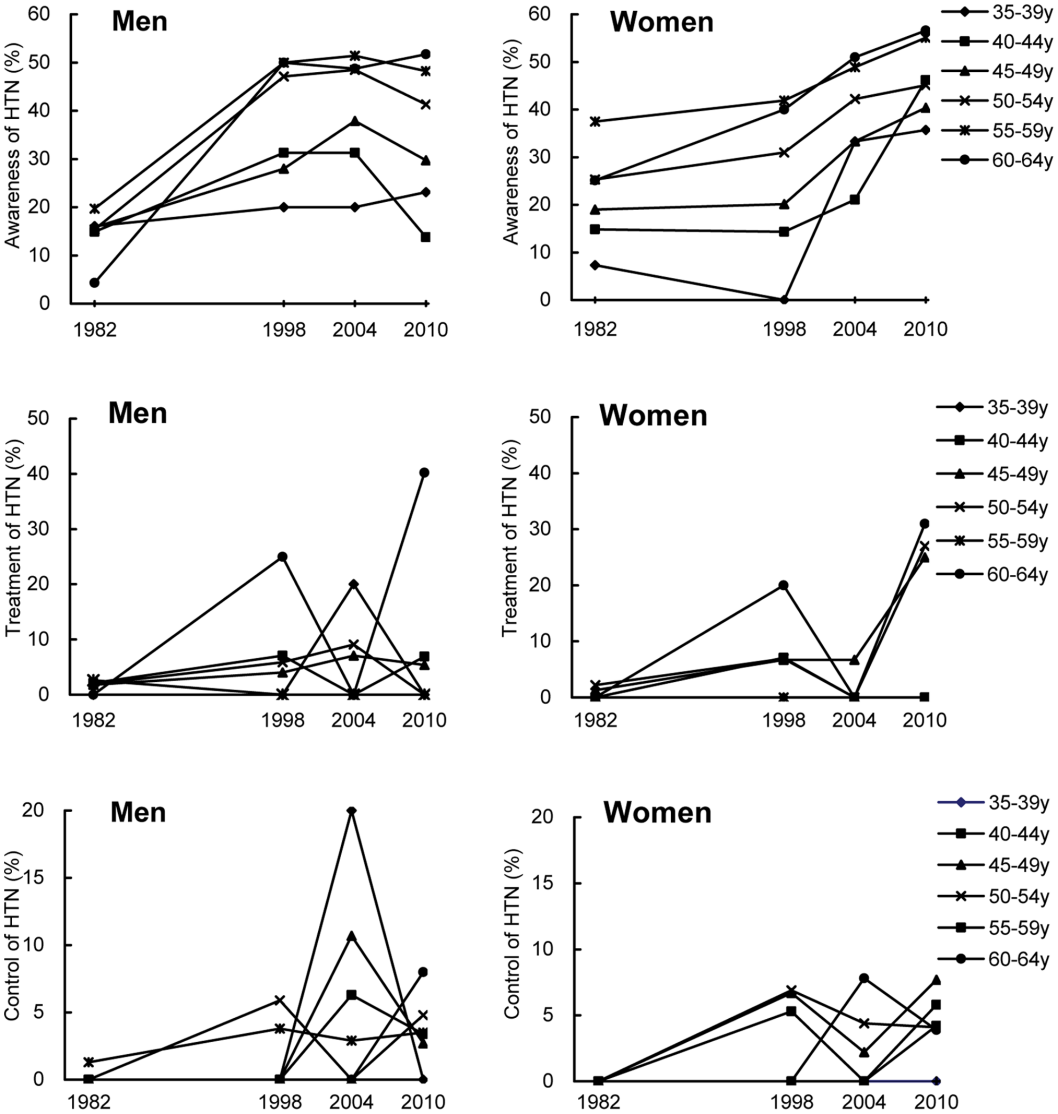
Trends in awareness, treatment, control of HTN among adults in different sex and age group, 1982 to 2010.

Awareness, treatment and control of HTN are presented in Table 4 and Figure 4. There was a significant improvement in awareness, treatment and control of HTN of over time (all P<0.01 for trend). The age- and sex- adjusted awareness increased from 16.8% in 1982 to 38.4% in 2010. Awareness increased significantly with age increasing among men and women in each year (all P<0.05 for trend), but was no difference between men and women (P>0.05). The age- and sex- adjusted rates of participants treated for their HTN with anti-hypertensive drugs increased from 1.0% in 1982 to 17.4% in 2010 (P<0.001 for trend). Treatment of HTN also increased with age among men and women in 2004 and 2010 (both P<0.05 for trend). There was no significant difference between men and women in treatment rates (all P>0.05), except for 45–49 years old adults in 2010. The age- and sex- adjusted control rate of HTN increased from 0.1% in 1982 to 3.5% in 2010 (P<0.01 for trend). No significant difference was found between control rates among men and women (P>0.05) and between different age groups (all P>0.05). Control rates among participants who treated their HTN were 12.8%, 35.3%, 27.5%, and 17.5% in 1982, 1998, 2004 and 2010, respectively, and showed no improvement during the past 28 years (P = 0.918 for trend).

**Table 4:** Awareness, treatment and control of HTN for adults aged 35 to 64 years from 1982 to 2010.

Characteristics	1982		1998		2004		2010		P value for trend
	n/N	% (95% CI)	n/N	% (95% CI)	n/N	% (95% CI)	n/N	% (95% CI)	
Awareness									
Overall*	129/651	16.8 (6.1–27.8)	74/219	27.1 (9.9–43.8)	186/423	37.8 (18.7–58.6)	426/916	38.4 (23.9–56.9)	<0.001
Men†	45/286	15.0 (4.8–26.9)	37/96	34.0 (18.3–49.7)	70/156	38.3 (14.1–64.9)	130/314	32.9 (17.9–56.8)	<0.001
Women†	84/365	18.8 (7.5–28.7)	37/123	19.8 (0.8–37.4)	116/267	37.3 (23.7–51.8)	296/602	44.3 (30.4–57.0)	<0.001
Treatment									
Overall*	8/651	1.0 (0.4–2.7)	18/219	7.0 (5.0–9.2)	61/423	16.4 (4.6–29.4)	249/916	17.4 (10.5–28.8)	<0.001
Men†	5/286	1.4 (0.5–3.4)	7/96	6.8 (5.8–7.8)	21/156	23.5 (4.9–46.0)	78/314	12.8 (8.0–22.6)	<0.001
Women†	3/365	0.6 (0.4–1.8)	11/123	7.1 (4.0–10.7)	40/267	8.7 (4.3–11.7)	171/602	22.3 (13.2–35.4)	<0.001
Control									
Overall*	1/651	0.1 (0.0–0.2)	8/219	2.1 (0.7–3.8)	17/423	4.9 (2.0–25.5)	43/916	3.5 (1.2–10.9)	0.009
Men†	1/286	0.1 (0.1–0.4)	2/96	1.3 (0.1–2.5)	6/156	7.6 (2.8–44.5)	15/314	3.0 (0.9–11.6)	0.001
Women†	0/365	0.0 (0.0–0.0)	6/123	2.9 (1.4–5.2)	11/267	1.9 (1.1–5.2)	28/602	4.0 (1.6–10.2)	0.047
Control in treatment									
Overall*	1/8	12.8 (4.5–30.0)	8/18	35.3 (21.8–62.9)	17/61	27.5 (12.5–40.3)	43/249	17.5 (9.9–29.1)	0.918
Men†	1/5	20.0 (7.2–44.5)	2/7	28.3 (12.1–46.8)	6/21	28.4 (17.4–56.6)	15/78	18.4 (11.5–28.0)	0.974
Women†	0/3	0.0 (0.0–0.0)	6/11	44.5 (13.6–73.9)	11/40	27.1 (13.2–45.9)	28/171	17.1 (8.7–30.8)	0.831

HTN, hypertension; 95% CI, 95% confidence interval.

* Age-and sex- adjusted percentages.

† Age- adjusted percentages for men or women. Adjustment was conducted with the 2000 Chinese National Census population by the direct method.

### Risk Factors for Prevalence, Awareness, Treatment, and Control of HTN

Results of Complex Samples General Linear Models analysis showed that age, BMI and having family history of HTN were positive associated with SBP and DBP (all P<0.001). No association was found between gender, level of education, smoking, drinking and BP level (all P>0.05).

Complex Samples Logistic Regression Models analysis on the risk factors for prevalence, awareness, treatment, and control of HTN are presented in Table 5. It showed that overweight (25.0≤BMI<30.0), obesity (BMI ≥30.0) and having family history of HTN were risk factors for HTN. The odds ratio (OR) and 95% confidence interval (95% CI) were 1.98 (1.44–2.74), 2.01 (1.79–5.11), and 2.05 (1.79–2.34), respectively, for overweight, obesity and having family history of HTN. Being underweight (BMI<18.5) was a protective factor for HTN (OR: 0.56; 95% CI: 0.38–0.80). Increasing age was a risk factor for HTN, and compared with participants aged 35–39 years, the ORs increased with age increasing, from 1.59 (95% CI: 1.39–1.83) of 40–44 years olds to 6.82 (95% CI: 5.44–8.55) of 60–64 years olds. Age and having a family history of HTN were associated with higher awareness of HTN. The ORs increased with age increasing, from 1.10 (95% CI: 1.03–3.34) of 40–44 years olds to 4.15 (95% CI: 1.13–15.21) of 60–64 years olds compared with 35–39 years olds. Family history of HTN gave an approximately two to threefold increase in awareness, treatment and control of HTN. Increasing age was also associated with higher treatment rate, and compared with participants aged 35–39 years, the ORs increased with age increasing, from 1.16 (95% CI: 1.09–5.11) of 40–44 years olds to 4.85 (95% CI: 1.75–31.16) of 60–64 years olds. Alcohol drinking was inversely associated with treatment and control of HTN, roughly halving prevalence. No association was found between gender, education level, marital status, smoking and prevalence, awareness, treatment and control of HTN (all P>0.05).

**Table 5:** Results of logistic regression analyses of risk factors for prevalence, awareness, treatment and control of HTN.

Variables	HTN (n = 4840)		Awareness (n = 1553)		Treatment (n = 1553)		Control (n = 1553)	
	OR (95% CI)	P value	OR (95% CI)	P value	OR (95% CI)	P value	OR (95% CI)	P value
Year								
1998*	1.00 (reference)		1.00 (reference)		1.00 (reference)		1.00 (reference)	
2004	1.69 (1.15–2.49)	<0.001	1.49 (1.05–2.33)	0.025	2.10 (1.07–4.13)	0.035	1.53 (0.35–6.65)	0.535
2010	2.25 (1.77–2.86)	<0.001	1.69 (1.12–2.56)	0.018	3.84 (2.91–5.06)	<0.001	1.30 (0.44–3.83)	0.598
Gender								
Men	1.00 (reference)		1.00 (reference)		1.00 (reference)		1.00 (reference)	.
Women	0.96 (0.79–1.16)	0.642	0.80 (0.41–1.56)	0.468	0.82 (0.24–2.75)	0.718	0.74 (0.16–3.41)	0.664
Age group								
35–39 y	1.00 (reference)		1.00 (reference)		1.00 (reference)		1.00 (reference)	.
40–44 y	1.59 (1.39–1.83)	<0.001	1.10 (1.03–3.34)	0.001	1.16 (1.09–5.11)	0.002	0.30 (0.02–4.53)	0.346
45–49 y	3.60 (2.83–4.57)	<0.001	1.60 (1.16–4.13)	0.001	1.60 (1.45–5.65)	0.001	1.11 (0.33–3.70)	0.851
50–54 y	4.06 (2.89–5.70)	<0.001	2.31 (1.79–6.73)	0.002	1.93 (1.47–7.88)	0.003	0.49 (0.05–5.34)	0.522
55–59 y	5.39 (4.30–6.74)	<0.001	3.69 (1.33–10.23)	0.017	3.24 (1.74–14.22)	0.007	0.46 (0.06–3.67)	0.426
60–64 y	6.82 (5.44–8.55)	<0.001	4.15 (1.13–15.21)	0.035	4.85 (1.75–31.16)	0.009	0.96 (0.04–24.38)	0.976
Marital status								
Married	1.00 (reference)		1.00 (reference)		1.00 (reference)		1.00 (reference)	.
Unmarried	0.29 (0.05–1.54)	0.129	0.53 (0.02–12.46)	0.661	2.69 (0.12–58.59)	0.491	0.12 (0.03–6.10)	0.999
Divorced, separate, widowed	1.46 (0.99–2.16)	0.055	0.74 (0.46–1.19)	0.183	0.88 (0.51–1.50)	0.599	1.06 (0.29–3.91)	0.919
Level of education								
Illiterate	1.00 (reference)		1.00 (reference)		1.00 (reference)		1.00 (reference)	.
Elementary	0.93 (0.62–1.41)	0.710	0.95 (0.63–1.44)	0.807	0.85 (0.49–1.45)	0.510	1.19 (0.39–3.66)	0.733
Middle School	0.89 (0.72–1.09)	0.228	0.97 (0.59–1.59)	0.888	1.13 (0.70–1.81)	0.592	0.94 (0.27–3.20)	0.908
High school and above	1.28 (0.89–1.83)	0.161	1.29 (0.50–3.34)	0.566	1.56 (0.48–5.06)	0.421	1.06 (0.31–3.70)	0.913
Family history of HTN								
No	1.00 (reference)		1.00 (reference)		1.00 (reference)		1.00 (reference)	.
Yes	2.05 (1.79–2.34)	<0.001	2.87 (1.71–4.82)	0.001	3.06 (2.13–4.41)	<0.001	2.46 (1.36–4.45)	0.007
Current cigarette smoking								
No	1.00 (reference)		1.00 (reference)		1.00 (reference)		1.00 (reference)	.
Yes	1.00 (0.76–1.33)	0.979	1.21 (0.42–1.87)	0.612	0.97 (0.33–2.87)	0.957	0.84 (0.31–2.31)	0.716
Current alcohol drinking								
No	1.00 (reference)		1.00 (reference)		1.00 (reference)		1.00 (reference)	.
Yes	0.88 (0.65–1.19)	0.373	0.97 (0.74–1.27)	0.821	0.45 (0.31–0.64)	<0.001	0.33 (0.13–0.84)	0.025
BMI category								
Normal (18.5≤BMI<25)	1.00 (reference)		1.00 (reference)		1.00 (reference)		1.00 (reference)	.
Underweight (BMI<18.5)	0.56 (0.38–0.80)	0.005	0.52 (0.13–2.17)	0.332	0.49 (0.13–1.88)	0.263	0.31 (0.04–2.24)	0.218
Overweight (25≤BMI<30)	1.98 (1.44–2.74)	0.001	1.04 (0.61–1.46)	0.762	1.09 (0.67–1.78)	0.686	0.85 (0.44–1.65)	0.599
Obese (BMI≥30)	2.01 (1.79–5.11)	0.001	1.08 (0.59–1.94)	0.790	1.16 (0.32–2.54)	0.826	1.09 (0.20–1.72)	0.798

HTN, hypertension; 95% CI, 95% confidence interval.

* No data about marital status, education level and family history of HTN in the 1982’s survey. The logistic regression analyses were conducted using data from 1998 to 2010.

## Discussion

This is the first study describing trends in BP levels and prevalence of HTN among rural people in Northwestern China. Four cross-sectional surveys were conducted on the same sites using similar methodology, and individuals in the same 35–64 years age range were included in the analysis. The results, adjusted for age and sex, showed that average BP levels in these populations significantly increased over the 28 year interval in both men and women and across age groups. From 1982 to 2010, mean SBP increased by 8.8 mm Hg, and mean DBP increased by 2.7 mm Hg. Prevalence of HTN increased from 18.4% in 1982 to 30.5% in 2010. Awareness, treatment and control of HTN among them displayed a similar increase pattern.

Our findings showing upward trends in mean SBP, mean DBP, prevalence, awareness, treatment and control of HTN are consistent with the study among Chinese adults from 1991 to 2009 by Xi and colleagues [19], and also consistent with a study among a rural population in Shandong Province of China from 1991 to 2007 [18]. In contrast with those two studies, we did not find an upward trend in pre-HTN; it remained relatively stable around 37%. The prevalence of HTN among rural adults in Northwest China, 30.5% in 2010, is closer to, even higher than, those of developed countries, such as 34.4% among adults aged 35 to 74 years in Switzerland between 1999 and 2009 [9], 29.0% among the USA adults aged older than 18 years between 1999 and 2008 [8] and 29.5% among the USA adults aged older than 20 years in 2009 to 2010 [4].

The upward trend in BP values and prevalence of HTN observed among rural population in Northwest China from 1982 to 2010 might be due to decrease in physical activity, changes in dietary habits, high salt intake, and increase in BMI and obesity. Lower BP is related to higher dietary fiber and potassium intake, and total or saturated fat intake correlates positively with BP [21]. Studies in China showed that between 1991 and 2006, average weekly physical activity among adults fell by 32% [22], high-strength physical activity decreased remarkably and the prevalence of overweight and obesity increased [18]. Further, there have been changes in the Chinese dietary pattern in the past decades. A study based on the China Health and Nutrition Survey showed that cereals, vegetables and dietary fiber intake has decreased, and meat, egg and oil intake significantly increased from 1989 to 2006 among Chinese adults [23], [24]. A study based on data from the China Health and Nutrition Survey (1989–1997) and the China National Nutrition Survey (1982 and 1992), also found that the intake of animal foods increased quickly after the economic reforms occurred in China and the Chinese have shift towards a high-fat, high-energy-density and low-fiber diet [25]. Study showed that people living in Northwestern China consumed significantly more sodium than people from the south of China [26]. BMI was a predictor of HTN [27]. Results of linear regression analysis in our study showed that BMI was positive associated with SBP and DBP, and logistic regression analysis revealed that overweight and obesity were risk factors for HTN. Our study also found the mean BMI, prevalence of obesity and overweight increased significantly from 1982 to 2010 (all P<0.001 for trend). Therefore, effective efforts should be made to improve the diet and lifestyle behaviors and to reverse the adverse trend in HTN prevalence among people in Northwest China.

Despite the upward trends, awareness, treatment, control, and control in treatment among our rural population of Northwest China, being 38.4%, 17.4%, 3.5% and 17.5%, respectively, in 2010, remain poor and are much lower than developed countries. In 2009 to 2010, awareness, management, control, and control in management rate of HTN were 74.0%, 71.6%, 36.8% and 64.4%, respectively, among the USA adults [4], and awareness, treatment and control rates among adults in Switzerland between 2004 and 2009 were 82.3%, 38.2% and 59.4%, respectively [9]. The treatment and control among our rural population were even lower than those among a rural population in Nepal, 23.5% for treatment and 9.5% for control [28], and lower than those among the partially representative Chinese adults in 2009 (19.8% for treatment, 4.4% for control, and 26.6% for control in treatment, respectively, in rural, and 28.0% for treatment, 9.5% for control, and 47.3% for control in treatment, respectively, in urban) [19].

Many factors contributed to poor awareness, treatment and control of HTN. Lack of BP measurement was associated with low awareness and treatment [29]. In developing countries, measurement of BP is not seen as a primary task by primary health-care workers and is not systematically done [13]. In the Chinese population, lack of awareness and monetary costs were the primary reasons for patients not taking antihypertensive medication [30], [31]. Patients’ poor medication adherence, poor beliefs about HTN and its treatment and the failure of health care providers to initiate or intensify drug therapy for patients were barriers to BP control [32]. Other factors, such as scarce health resources, poor health infrastructure, shortage of primary care physicians, shortage of effective models for hypertension management and effective performance monitoring and feedback systems, and shortage of logical reimbursement models are also barriers to BP control [32], [33], [34], [35]. Studies have also shown that the implementation of hypertension guidelines in clinical practice is often inadequate [36]. Therefore, strategies and education programs should be initiated to enhance patients’ and physicians' awareness and improve the health care system to facilitate the effective control of HTN in the rural Northwest China. And efforts should focus on enhancing treatment effectiveness. Combined behavioral telephone intervention and home BP monitoring have been found to be effective to promote BP control by a randomized trial conducted in Duke University Health System primary care clinics in the USA [37]. Also, messages sent to mobile phones are another method to control HTN [38], [39], [40] and could be considered. In recent years, the New Rural Cooperative Medical System, which aims to provide health insurance to rural population and to correct distortions in rural primary care, has been widely establishing in China and has provided some financial risk protection for individuals in rural China [41] as well as improved the health-care utilization of rural elders [42]. To cover the outpatient and inpatient services of non-communicable diseases, such as hypertension and diabetes, into reimbursement of the New Rural Cooperative Medical System may improve the rural population’s affordability and utilization of HTN management services and improve the control of HTN.

Moreover, our study has shown that BP level and prevalence, awareness and treatment of HTN were positively associated with increasing age, BMI and having family history of HTN. These findings suggest that older, obese people and people with family history of HTN are at high risk of HTN and should be the important target population of prevention and control of HTN, and efforts to improve BP control in younger adults should focus on raising awareness and treatment.

Our study has some potential limitations. First, low treatment of HTN and relatively small sample size in each age and sex group made it impossible to perform trend analysis on control in treatment of HTN in age/sex subgroups. Second, in the four surveys in this analysis, the diagnosis of HTN is based on the BP measurement at a single visit on the examination day, and not as recommended for clinical practice on three or more measurements at intervals of two weeks. BP measurement at a single visit usually overestimates HTN prevalence and underestimates control rate [8], [19], [43]. However, BP measurement at a single visit is often used in epidemiologic studies. Third, dietary intakes and physical activity level are associated with BP level and hypertension [21], and economic level is main impact factors for treatment and control rate of HTN [30], [31]. However, the four surveys in this analysis had no comparable data on dietary, physical activity and economic level of participants. It was a disadvantage of the study and limited the detailed analysis on the causes of trends in prevalence, awareness, treatment and control of HTN. A major increase in mean SBP occurred between 2004 and 2010 surveys, whereas DBP remained quite stable. Standard mercury sphygmomanometers were used for all four surveys. With the exception of the 2010 survey, blood pressure was based on the average of two, rather than three measurements. As a check, we examined the differences between the averages of the first two readings versus those of all three in the earlier surveys and found them substantially the same. So the measurements were consistent. The difference in SBP and DBP change trend between 2004 and 2010 may be accounted for a different response of SBP and DBP to diet, physical activity and other lifestyle changes and/or social and economic changes. However, we have no enough data to demonstrate this hypothesis and it is needed further study. Despite these limitations the study is the first population-based study in China to track hypertension in a fixed geographic area over 28 year period in four surveys. The size of each study is large enough to elicit the main effects at each time point and establish trends.

## Conclusions

The mean population BP and prevalence of HTN among middle-aged and older adults in the rural areas of Hanzhong have increased between 1982 and 2010. However, awareness, treatment and control rates of HTN remain unacceptably low. Public health programs and practical strategies are required to improve prevention, management and control of HTN among the rural population in Northwest China. In particular, attention should be given to the elderly and obese, and to those with a family history of hypertension, while raising awareness and treatment among younger adults.
